# Investigating the mechanism of aluminum resistance conferred by aluminum resistance protein 1

**DOI:** 10.1002/pro.70405

**Published:** 2025-12-22

**Authors:** Pavlo Stehantsev, Artem Stetsenko, Pavla Herynková, Andrei Zupnik, Carsten Takens, Olga Zimmermannova, Hana Sychrova, Albert Guskov

**Affiliations:** ^1^ Groningen Biomolecular Sciences and Biotechnology Institute University of Groningen Groningen the Netherlands; ^2^ Laboratory of Membrane Transport Institute of Physiology CAS Prague Czechia

**Keywords:** ALR, aluminum resistance, CorA, membrane protein, membrane transport

## Abstract

Aluminum does not play any role in essential biological processes, but in acidic conditions it becomes biologically reactive and highly toxic even at low concentrations. Eukaryotes have developed numerous strategies for detoxification, one of which relies on membrane proteins. Fungi possess so‐called aluminum resistance proteins (Alr1/2p), which are distant homologs of CorA Mg^2+^‐channels but are claimed to confer resistance to aluminum. However, the mechanism behind this resistance remains poorly understood. In this study we aimed to conduct a thorough functional and structural characterization of Alr1p from *Pichia pastoris* (PpAlr1p). Our results confirm that PpAlr1p similar to other members of the CorA family functions as a pentamer and can mediate Zn^2+^ and Cd^2+^ transport across the membrane independently from a pH gradient. Studies on the truncated version of PpAlr1p define the core functional unit of CorA proteins but pinpoint that the intracellular domain is important for the correct localization. Competitive uptake assays with Al^3+^ showed aluminum to activate the transport of divalent cations in PpAlr1p. In addition, the growth assay of the *ALR2* knock‐out *Saccharomyces cerevisiae* strain indicates some role of Alr2p in promoting tolerance to Ni^2+^. Together, these results provide the functional and structural characterization of a eukaryotic CorA‐like aluminum resistance protein, highlight the role of its N‐terminal domain in proper cellular localization and define the functional core of the CorA family.

## 1 INTRODUCTION

Aluminum is the third most abundant element and the most abundant metal on the Earth (Moeller et al., 1984), normally present in a nontoxic form as silicates or oxide minerals at neutral to slightly acidic conditions (pH >5.5). However, its toxicity and bioavailability greatly increase in acidic conditions (Delhaize & Ryan, 1995; Exley & Birchall, 1992). Aluminum plays no role in any essential biological processes, but under acidic conditions, even small amounts of the soluble ion exhibit high biological reactivity and toxicity. Aluminum is a highly charged small cation that differs significantly from biologically essential ions (MacDiarmid & Gardner, 1998). Al^3+^ readily binds to organic acids, proteins, and lipids, competing with vital microelements such as Mg^2+^, Ca^2+^, and Co^2+^, causing their deficiency (Delhaize & Ryan, 1995; Kiss & Hollósi, 2001; MacDiarmid & Gardner, 1998; Martin, 1986; Poschenrieder et al., 2008; Routa et al., 2001).

In response to the toxic impact of aluminum, plants have evolved various resistance mechanisms. The tolerant species showcase increased levels of inorganic substances (mono‐/divalent cations) and organic acids (malate, succinate, citric acid), facilitating the formation of harmless aluminum complexes (Delhaize & Ryan, 1995; Kochian et al., 2004; Routa et al., 2001).

Alr proteins, Alr1p and Alr2p, reside in the plasma membrane of fungi and are proposed to play a role in aluminum tolerance mechanisms. Alr1/2 proteins belong to the eukaryotic branch of the CorA family of magnesium‐uptake systems. Besides Mg^2+^, Alr1p was proposed to transport divalent ions: Cd^2+^, Ca^2+^, Co^2+^, Mn^2+^, Ni^2+^, and Zn^2+^ with different levels of efficiency (Hanner et al., 2019; Kern et al., 2005; Lim et al., 2011; MacDiarmid & Gardner, 1998) and confer resistance to trivalent Ga^3+^ and Al^3+^ (MacDiarmid & Gardner, 1998). Among the CorA family only Alr1/2p and Mrs2‐10 (another eukaryotic CorA member from *Arabidopsis thaliana*; Ishijima et al., 2018) are proposed to be affected by Al^3+^ (Ishijima et al., 2018; MacDiarmid & Gardner, 1998). *ALR1* encodes the major magnesium uptake protein in strains of *Saccharomyces cerevisiae*, and *ALR2* appears to be a minor functional equivalent (MacDiarmid & Gardner, 1998). Alr1 and Alr2 exhibit significant sequence similarity with each other (approximately 70%) while displaying a low degree of similarity with other CorAs (MacDiarmid & Gardner, 1998). Although the CorA family shows in principle quite low overall sequence identity, the proteins reveal structural conservation: the symmetrical pentamer with a membrane‐spanning central pore, large cytoplasmic N‐terminal domain, and two transmembrane helices featuring a conserved motif acting as a selectivity filter connected by a periplasmic loop (Eshaghi et al., 2006). The peculiarity of Alr1p is that it has an even larger N‐terminal cytoplasmic domain (~400 extra residues long) with at least partially structurally disordered organization (Lee & Gardner, 2006). This region is unique for Alr1/2p and is much shorter in other CorAs. Alr1p has the ability to form heteromers with Alr2p, which potentially can modulate Alr1p activity (Wachek et al., 2006). Interestingly, it seems that during evolution, the mechanism of cation transport within the CorA family has been tailored to the needs and environmental niche of organisms.

The most detailed description of a transport mechanism is available for CorA from *Thermotoga maritima* (*Tm*CorA) (Eshaghi et al., 2006; Payandeh & Pai, 2006). It is generally accepted that when *Tm*CorA is exposed to normal or high levels of Mg^2+^, it resides in a closed conformation, preventing ion conduction, and this state is stabilized by the interactions between the N‐terminal domains of neighboring subunits and bound magnesium ions at the Mg^2+^‐binding sites (Eshaghi et al., 2006). After the drop in Mg^2+^ concentration, bound ions escape the binding sites, leading to enhanced flexibility of N‐terminal domains eventually leading to an asymmetric state of *Tm*CorA, facilitating ion conduction through the central pore (Matthies et al., 2016; Nemchinova et al., 2021). The structure of CorA from *Methanocaldococcus jannaschii* (*Mj*CorA) displayed a similar domain arrangement, albeit with alternative magnesium binding locations—termed grooves (Guskov & Eshaghi, 2012). In magnesium‐depleted conditions, *Mj*CorA was reported to have an asymmetric assembly (Cleverley et al., 2015). However, this observation is somewhat ambiguous due to the low resolution and intransparency of a sample preparation. Furthermore, we observed a symmetric *Mj*CorA in an Mg^2+^‐free condition (unpublished data). The zinc‐specific transporters of the CorA family ZntB presumably employ a charge inversion for the transport, without transitioning to an asymmetric state (Gati et al., 2017).

Up to date the information on the possible transport mechanism in Alr1/2p is extremely limited. It was proposed that Alr1/2p are likely channels that act as proton‐coupled symporters, and mediate both the influx and efflux of magnesium (Lee & Gardner, 2006; Liu et al., 2002; MacDiarmid & Gardner, 1998). However this was never confirmed and also the possible mechanism of the Alr‐promoted aluminum resistance remained elusive.

To answer these questions, we performed a functional characterization of a truncated version of the Alr1p (residues 352–725; hereafter Alr1p‐T) from *Pichia pastoris* and solved a low‐resolution structure using single‐particle cryo‐electron microscopy. PpAlr1p‐T assembles into a pentamer and defines the functional unit of CorA proteins, which is capable of transporting Zn^2+^ in pH‐independent manner. Fluorescence microscopy indicates the importance of the N‐terminal domain for protein localization, whereas competitive transport uptakes of divalent cations in the presence of aluminum indicate the activating role of PpAlr1p‐T. Additionally, the growth assay of the *ALR2* knock‐out *S. cerevisiae* strain indicated some role of ScAlr2p in promoting tolerance to Ni^2+^.

## 2 MATERIALS AND METHODS

### 2.1 Alr1p cloning and expression

The *Escherichia coli* codon‐optimized truncated *ALR* gene from *Komagataella phaffii* (*P. pastoris*) (residues 352–725 aa) incorporated into the pET‐28(+) vector was ordered from GenScript, and transformed (heat‐shock at 42°C for 45 s) into the chemically competent *E. coli* C43(DE3) cells. The transformed cells were cultivated in 1 mL Lysogeny broth (LB) for 45 min and grown on plates of LB (10 g/L Bacto tryptone, 5 g/L Bacto yeast extract, 10 g/L NaCl) supplied with 50 μg/mL kanamycin, at 37°C, overnight. The next day, a few single colonies were picked, and grown in 5 mL of liquid LB with 50 μg/mL kanamycin. Further the construct was isolated and sent for analysis (DNA sequencing).

### 2.2 Large‐scale protein production and purification

The transformed *E. coli* cells were pre‐cultured in 100 mL of LB supplied with 50 μg/mL kanamycin in a 1 L baffled flask at 37°C, 210 rpm, overnight. The next day, for large‐scale production, 2 L of LB in a 5 L baffled flask was diluted with the pre‐culture to the starting OD_600_ of 0.1. The cells were grown up to an OD_600_ of 0.6 at 37°C, 210 rpm. For induction of protein production, the cells were provided with isopropyl β‐D‐1‐thiogalactopyranoside (IPTG) to a final concentration of 0.1 mM for 12 h and the temperature was set to 18°C. Further, the cells were collected by centrifugation (15 min, 7500 g, 4°C), re‐suspended in Buffer A (50 mM Tris–HCl pH, 8.0; 300 mM NaCl), supplied with 0.1 mg/mL deoxyribonuclease (Sigma‐Aldrich) and 1 mM MgSO_4_, and broken in two passages (25 kPsi, 4°C) by high‐pressure disruption in a Maximator High Pressure Homogenizer Type HPL6 (Maximator GmbH). The broken cell suspension was supplied with 1 mM phenylmethylsulfonyl fluoride (Roche) protease inhibitor and centrifuged (30 min, 10,000 g, 4°C) to remove the heaviest cell debris. To spin down the MVs, the supernatant was centrifuged at ultra‐speeds (120 min, 194,000 g, 4°C) and settled MVs were resuspended in buffer B (50 mM Tris–HCl, pH 8.0; 300 mM NaCl; 10% (vol/vol) glycerol), flash frozen in liquid nitrogen and stored at −80°C.

The frozen MVs were thawed at room temperature and re‐suspended in Buffer C (50 mM Tris–HCl pH 8.0; 300 mM NaCl; 0.5 mM MgCl_2_; 1% [w/v] n‐dodecyl‐β‐D‐maltopyranoside [DDM, Anatrace]) for 1 h, 4°C, nutating. The suspension was spun down at ultra speeds (30 min, 236,000 g, 4°C) to isolate soluble particles and incubate them with Ni^2+^‐sepharose resin in a 10 mL disposable column (Bio‐Rad). The Ni^2+^‐sepharose resin in a volume of 0.5 mL was equilibrated with 10 times CV of dH_2_O for 1 h, 4°C, nutating. After 1 h, the unbound material was let to flow through the column, washed with Buffer D (50 mM Tris–HCl pH 8.0; 300 mM NaCl; 0.5 mM MgCl_2_; 40 mM imidazole; 0.02% [w/v] DDM). The protein was eluted with Buffer E (50 mM Tris–HCl, pH 8.0; 300 mM NaCl; 0.5 mM MgCl_2_; 500 mM imidazole; 0.02% [w/v] DDM) in three fractions of 250, 750, and 500 μL, respectively. The size‐exclusion chromatography column Superdex 200 10/300 (GE‐Healthcare) was equilibrated with Buffer F (50 mM Tris–HCl, pH 7.4; 300 mM NaCl; 0.5 mM MgCl_2_; 0.02% [w/v] DDM), and further the second elution fraction of 750 μL was purified via the column. The protein‐containing fractions were combined and concentrated to the required concentrations.

### 2.3 Lipids preparation

Dissolved in chloroform polar lipids of *E. coli* and egg phosphatidylcholine in a ratio of 3:1 (w/w) were dried in an evaporator and resuspended in a buffer of 50 mM KPi, pH 7.5 to a concentration of 20 mg/mL. Large unilamellar vesicles (LUVs) were obtained by three cycles of freeze–thaw, aliquoted, and stored in liquid nitrogen (LN).

### 2.4 Reconstitution into proteoliposomes

The aliquot of LUVs in a concentration of 20 mg/mL was extruded 11 times through a 400 nm pores filter (Avestin), diluted to 4 mg/mL in 70 mM HEPES, pH 7.5 and gradually destabilized with Triton X‐100 to the condition of the preformed liposomes. Further, the liposomes were combined with purified PpAlr1p‐T at a ratio of 1:250 (w/w, protein/lipid). In 0.5 h, the subsequent addition of absorbent (Bio‐beads SM‐2, Bio‐Rad, USA; 40 mg/mL) was performed for detergent removal (four times after 0.5, 1, 2 h, and overnight). The next day, the proteoliposomes were collected by centrifugation (20 min, 347,500 g, 4°C), resuspended in 70 mM HEPES, pH 7.5 to 20 mg/mL and directly used for experiments or frozen and stored in LN_2_.

### 2.5 Fluorescence transport uptakes

To test the ability of PpAlr1p‐T to transport divalent cations we encapsulated the fluorophore FluoZin‐1 (ThermoFisher, USA) sensitive to Zn^2+^, Cd^2+^, Co^2+^, and Ni^2+^. We opted for this dye due to its excellent signal and robustness. The fluorescence signal was measured upon translocation of the substrate into the proteoliposome. The proteoliposomes were mixed with FluoZin‐1 to a final concentration of 5 μM and encapsulated into the liposomes by three freeze–thaw cycles and subsequent extrusion through the 400 nm pores filter. Further, the suspension was passed through a size‐exclusion chromatography column filled with 1 mL Sephadex G‐75 equilibrated with 70 mM HEPES, pH 7.5, and the proteoliposomes were collected by ultracentrifugation (20 min, 347,500 g, 4°C). The isolated proteoliposomes were diluted by 70 mM HEPES, pH 7.5 to a total volume of around 50 μL per 4 mg of proteoliposomes.

Transport assays were performed by 400 μg of proteoliposomes in 1 mL 70 mM HEPES, pH 7.5 at 20°C, stirring at 350 rpm. An experiment was conducted to measure the fluorescence time course in a 1 mL cuvette with a stirrer, utilizing an excitation wavelength of 490 nm and an emission wavelength of 520 nm. The translocation of the substrate is induced at 55 s by the addition of studied ions and measured for 300 s. The liposomes without incorporated protein were used as a control. All the measurements were repeated at least three times.

### 2.6 Fluorescence microscopy

Microscopy photos of *S. cerevisiae* BY4741 *alr2Δ* cells expressing the *P. pastoris* full‐length Alr1p (PpAlr1p‐F) and Alr1p‐T protein variants tagged with GFP at the C‐terminus (both expressed from pGRU1 plasmid under the control of *ScNHA1* promoter).

Further the cells were grown in YNB media to the exponential phase (OD_600_ = 0.3–0.5) and visualized with an Olympus BX53 microscope and Olympus DP73 camera (Olympus, Tokio, Japan). A cool LED light source with 460 nm excitation and 515 nm emission was used for fluorescence images. Nomarski optics was used for whole‐cell images.

### 2.7 Cryo‐EM sample preparation

The purified protein was concentrated to a final concentration of ~4.7 mg/mL with the Vivaspin Turbo 4 Concentrator (100 kDa cutoff size, Sartorius). Holey grids (Quantifoil Cu R1.2/1.3, 300 mesh) were glow‐discharged for 4 s and covered with the sample aliquots of 2.7 μL. Further, they were blotted for 4 s using FEI Vitrobot Mark IV, and frozen in liquid ethane. The grids were measured in Talos Arctica TEM (Thermo Fisher) with a K2 detector. See Table 1 for details.

**TABLE 1:** Cryo‐EM data collection.

Data collection and processing	
Magnification	130,000
Voltage (kV)	200
Electron exposure (e−/Å^2)	60
Energy filter (eV)	20
Defocus range (μm)	−1.0 – −2.0
Pixel size (Å)	1.022
Symmetry imposed	C5
Micrographs collected (#)	1382
Initial particle images (#)	100,893
Final particle images (#)	25,826
Map resolution (Å) FSC = 0.143	6.18

### 2.8 Cryo‐EM data processing, model building, and refinement

Patch motion correction, CTF estimation, particle picking, 2D classification, Ab‐initio reconstruction, and 3D refinement were performed in CryoSPARC (Structura Biotechnology Inc.). The model was generated with AlphaFold (Jumper et al., 2021; Varadi et al., 2022) and manually placed into density with Chimera, followed up by real space refinement in Phenix (Liebschner et al., 2019). PyMol (Schrödinger, 2010) and UCSF Chimera (RBVI) were used for structure interpretation and visualization.

### 2.9 Construction of *alr2Δ* single deletion in *S. cerevisiae*

The *ALR2* gene was deleted by homologous recombination using the Cre‐loxP system with the KanMX marker gene (Güldener et al., 1996) in *S. cerevisiae* BY4741 strain (*MATα his3Δ1 leu2Δ0 met15Δ0 ura3Δ0*). The used oligonucleotides are listed in Table S1, Supporting Information. The cells were transformed by electroporation, and further cassette integration and the absence of the *ALR2* gene were confirmed by polymerase chain reaction (PCR). *E. coli* XL1‐Blue (Stratagene) was used for plasmid amplification.

### 2.10 Cloning of full and truncated versions of *P. pastoris* ALR1 cDNA

For the overexpression of the pALR1‐F (full‐version *Pp*Alr1p) and pALR1‐T (residues 352–725 of *Pp*Alr1p) and their GFP tagged versions (pALR1‐F‐GFP and pALR1‐T‐GFP, respectively), corresponding oligonucleotides for amplification were designed with 40 nt flanking regions (Table S2) and were amplified by PCR using the *P. pastoris* genomic DNA as a template.

The obtained PCR fragments were cloned by homologous recombination into multicopy plasmids based on YEp352 and pGRU1 (enabling GFP tagging of the ORF at the 3′ terminus) behind the constitutive *S. cerevisiae* NHA1 promoter containing the URA3 selection marker. It resulted in the plasmids pALR1‐F, pALR1‐T, pALR1‐F‐GFP, and pALR1‐T‐GFP, respectively. All constructs were selected on YNB w/o Uracil plates (Difco; prepared by adding 2% [w/v] agar). Further, integration of DNA fragments was confirmed by PCR, and the correct sequence of all constructs and proper GFP tagging were confirmed by sequencing. For the negative control the empty YEp352 (Hill et al., 1986) was used.

### 2.11 Drop test

For the drop tests, the cells were pre‐grown at 30°C on yeast nitrogen base plates (YNB; Difco; pH 3.5), prepared by adding 2% (w/v) agar. Further, the cells were suspended in sterile water to OD_600_ = 0.6 (Spekol 211, Carl Zeiss). Serial 10‐fold dilutions of cell suspensions were prepared and spotted on YNB with increasing concentrations of alkali‐metal cation salts. The supplemented salts and their concentrations were chosen according to (MacDiarmid & Gardner, 1998), and individually indicated for each condition.

## 3 RESULTS

### 3.1 The truncated PpAlr1p construct

**FIGURE 1:**
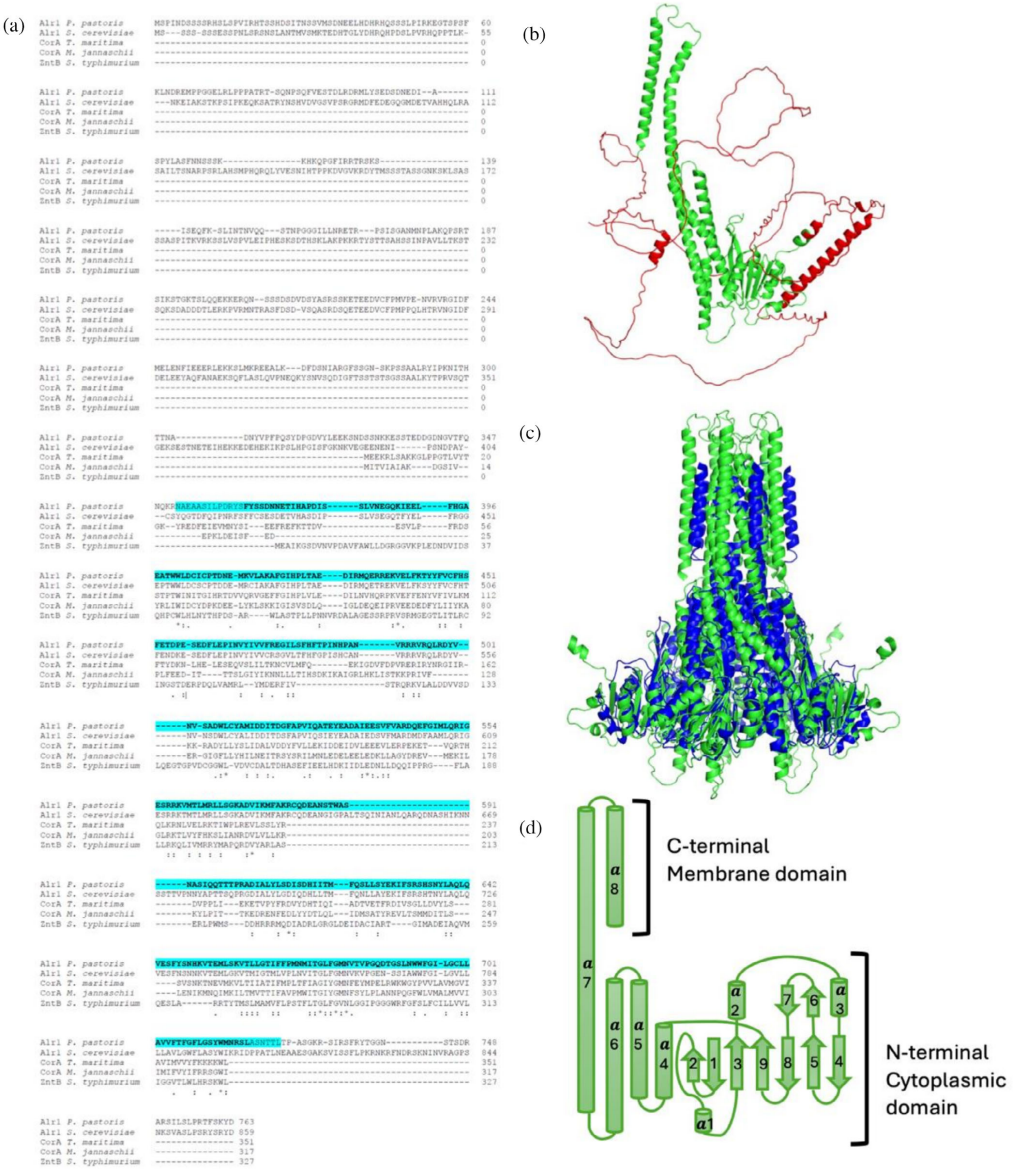
Comparison to the homologs and design of the truncated PpAlr1p version. An asterisk indicates amino acid identity; a dot indicates the exchange with a homologous amino acid (a). Bold text shows predicted non‐disordered regions in the full‐length Alr1p *Pichia pastoris* by DISOPRED2, where cyan color highlights the designed truncated version of PpAlr1p (b). An AlphaFold prediction of a protomer of PpAlr1p, red color indicates the truncated region, unfolded parts correlate well with DISOPRED2 prediction. (c) Comparison of PpAlr1p‐T (green) with TmCorA (dark blue). (d) The secondary structure diagram of PpAlr1p.

The full‐length PpAlr1p includes regions lacking ordered three‐dimensional structure, making it problematic for expression both in *E. coli* and *P. pastoris*. These disordered regions were predicted by DISOPRED2 (Ward et al., 2004) (Figure 1a, bold) and based on this analysis we designed a truncated version of Alr1p that begins with the residue 352 and ends at the position 725 (Figure 1a, cyan).

The truncation leads to a decrease of total PpAlr1p's length from 763 residues down to 373 (reducing Mw twice: 43 vs. 86 kDa), making it similar to prokaryotic CorAs (*Tm*CorA, *Mj*CorA, and *Ec*ZntB) by sequence, size, and apparently structure (Figure 1d). To test whether this truncated version can substitute the functional core of CorA‐related proteins we performed the structural and functional analysis.

### 3.2 PpAlr1p structure

**FIGURE 2:**
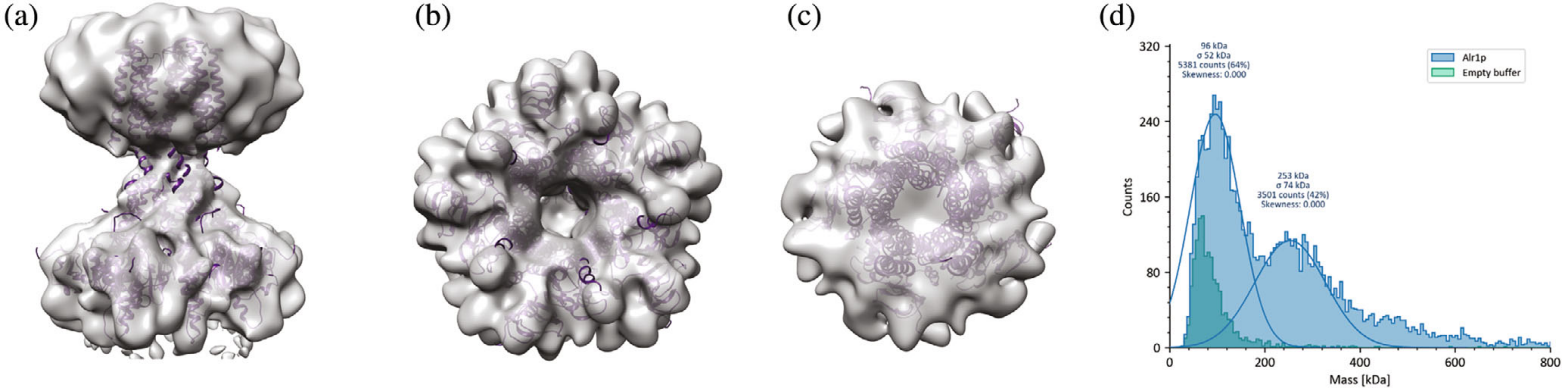
The coarse model (purple) of PpAlr1p‐T fitted into the 3D reconstruction of PpAlr1p obtained in DDM shows a pentameric assembly (light purple). (a) Side view, (b) cytoplasmic view, and (c) periplasmic view. The weak density between the cytoplasmic and transmembrane parts is indicative of intrinsic flexibility in this region. (d) The presence of pentameric PpAlr1p‐T in the solution as measured by mass‐photometry.

Using single‐particle Cryo‐EM, the truncated version of the PpAlr1p solubilized in detergent was resolved at an overall resolution of 6.18 Å with C5 symmetry applied (Figure 2). The quality of the map was sufficient to observe the pentameric arrangement and to place an AlphaFold (Jumper et al., 2021; Varadi et al., 2022) generated PpAlr1p model. The reconstruction in C1 is also indicative of the pentamer and importantly the mass photometry experiments also revealed the pentameric arrangement of PpAlr1p in solution (Figure 2d). This pentameric arrangement is the same as observed for other members of the CorA family despite a low sequence identity (Eshaghi et al., 2006; Gati et al., 2017; Guskov & Eshaghi, 2012; Nordin et al., 2013).

Due to the low resolution a more detailed analysis is not feasible at this point and so far, all of our attempts to improve the resolution have failed. Nevertheless, this low‐resolution structure confirms that even distant homologs of the CorA family maintain a pentameric arrangement. Furthermore, since the sample was prepared with Mg^2+^‐rich conditions we believe we observed a closed non‐conductive state of PpAlr1p.

### 3.3 Functional characterization

**FIGURE 3:**
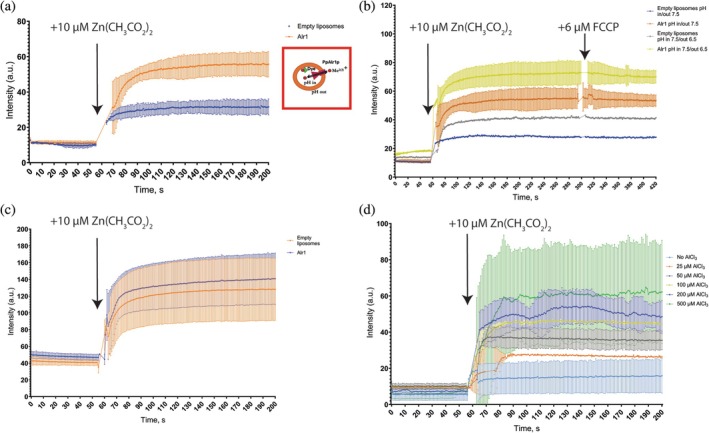
PpAlr1p‐T transport assays in proteoliposomes. The red box in panel (a) describes schematically the experiment: PpAlr1p‐T (magenta) reconstituted into a liposome (orange) assayed for transport of cations (Me^2(3)+^ [red spheres]) by monitoring the fluorescence of a dye (green). In all experiments cations were added at 55th second. (a) Uptake of Zn^2+^ as monitored by fluorescence increase of fluorophore FluoZin‐1. (b) Effect of a membrane potential and proton gradient on PpAlr1p‐T transport of Zn^2+^ as observed by dequenching of FluoZin‐1 dye fluorescence inside the proteoliposomes. About 6 μM of FCCP was added after 300 s. (c) Inability of PpAlr1p‐T to transport Al^3+^. Measured by morin dye. (d) Normalized inhibited PpAlr1p‐T transport of Zn^2+^ in the presence of various concentrations of AlCl_3_. Measured by morin dye, aluminum was added right before zinc.

To verify that the truncated version of PpAlr1p is functional we performed fluorescent transport assays we developed earlier (Stetsenko & Guskov, 2020). We assayed the transport via Zn^2+^ uptakes since the signal is much more pronounced and reliable compared to Mg^2+^ detecting dyes Fluozin‐3 or Mag‐fura 2 (Stetsenko & Guskov, 2020). In brief, freshly purified PpAlr1p‐T was reconstituted into proteoliposomes, and its transport capability was probed by monitoring fluorescence upon binding of transported cations to the FluoZin‐1 dye encapsulated inside the proteoliposomes. PpAlr1p‐T readily transports Zn^2+^ (Figure 3a), similarly to other CorAs (Gati et al., 2017; Stetsenko & Guskov, 2020).

In contrast to CorA from *T. maritima* and *M. jannaschii* (Stetsenko & Guskov, 2020), the transport by PpAlr1p is not affected by membrane potential (Figure 3b). In addition, it also seems to be pH‐independent (Figure 3b). This suggests PpAlr1p may rather function as a passive channel for divalent cations, rather than a proton‐coupled symporter as proposed for ScAlr1p.

As per the transport of Al^3+^, we failed to detect any significant increase in signal using morin dye in our experimental setup (Figure 3c). Previously we observed similar behavior by *Tm*CorA, *Mj*CorA, and *Ec*ZntB (Stetsenko & Guskov, 2020), so we cannot rule out that this dye is simply incompatible with our setup, especially since we do not have a rigid positive control.

Since morin dye failed to show any significant transport in Al^3+^ uptakes, we checked whether aluminum could inhibit zinc transport during the experiment (Figure 3d). Interestingly, an increase in aluminum concentration facilitates the transport of divalent cations (Figure 3d), up to concentrations when aluminum becomes damaging for proteoliposomes (at more than 100 μM AlCl_3_; shown by sharp peaks in curves of 200 μM (light blue) and 500 μM (green) AlCl_3_ in the Figure 3d) causing their instability.

### 3.4 The N‐terminal part of PpAlr1p is important for the plasma‐membrane targeting

**FIGURE 4:**
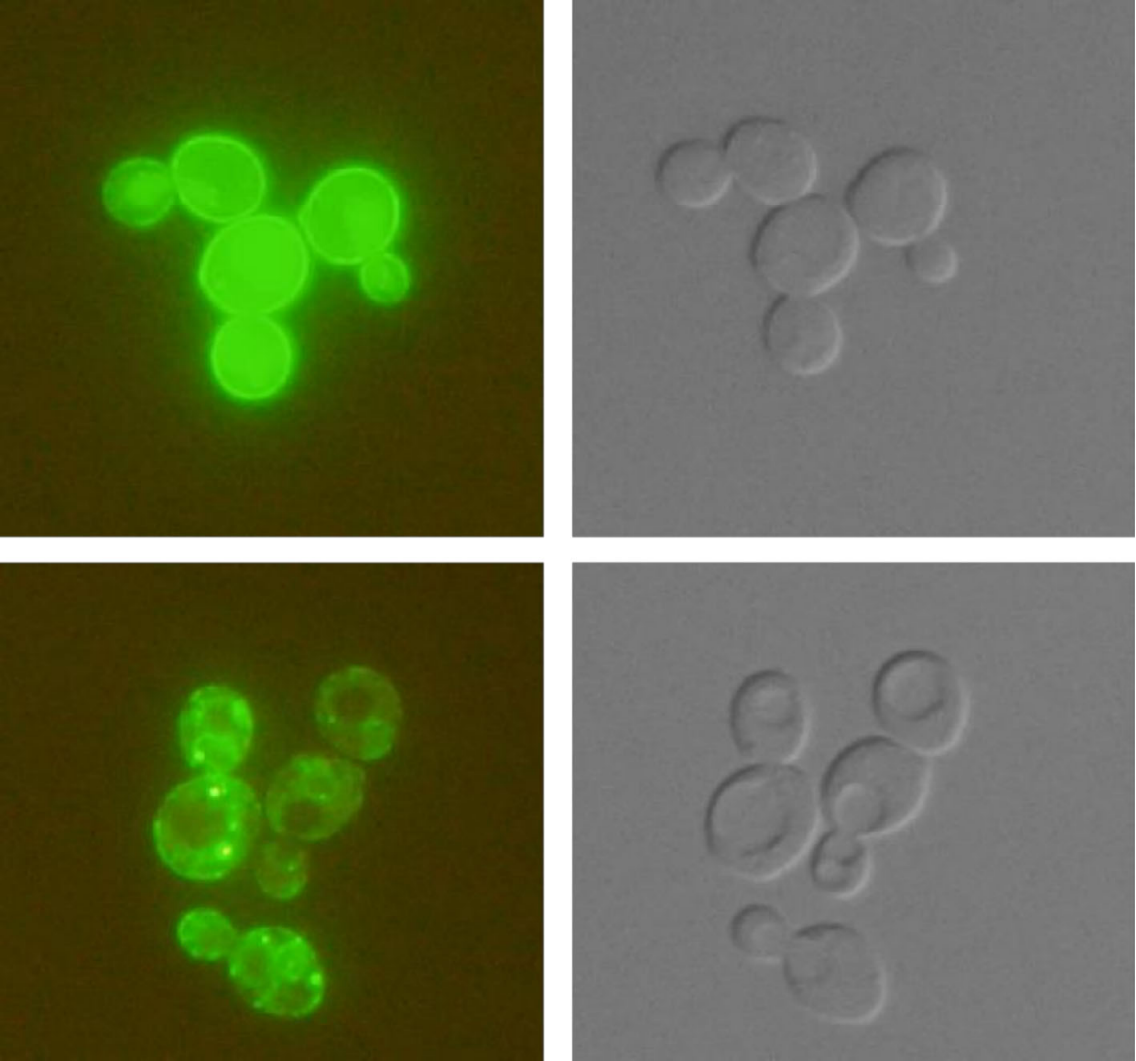
Localization of GFP‐tagged *Pp*Alr1‐full‐length (top) and *Pp*Alr1‐truncated (bottom) proteins expressed in *Saccharomyces cerevisiae*. The cells were analyzed with fluorescence (left) and Nomarski (right) microscopy.

To check the importance of the N‐terminal hydrophilic part of *Pp*Alr1p for protein localization and function, the full and truncated versions of *Pp*Alr1p (tagged with GFP at the C‐terminus) were expressed (from the multi‐copy plasmids under the control of the weak and constitutive *ScNHA1* promoter) in the *S. cerevisiae* strain lacking the *ALR2* gene (BY4741*alr2Δ*). As shown in Figure 4, fluorescence and Nomarski microscopy pictures of cells show clearly that the truncated version, albeit functional in vitro when reconstituted in proteoliposomes (Figure 3), has serious localization issues in vivo, as the majority of *Pp*Alr1‐T‐GFPp seems to be retained in the secretory pathway, and does not reach the plasma membrane as the full version of *Pp*Alr1‐F‐GFPp. This highlights the importance of the unstructured N‐terminal domain, which at least ensures the correct plasma‐membrane targeting of *Pp*Alr1p.

### 3.5 Lack of Alr2 in *S. cerevisiae* results in sensitivity to Ni^2+^

**FIGURE 5:**
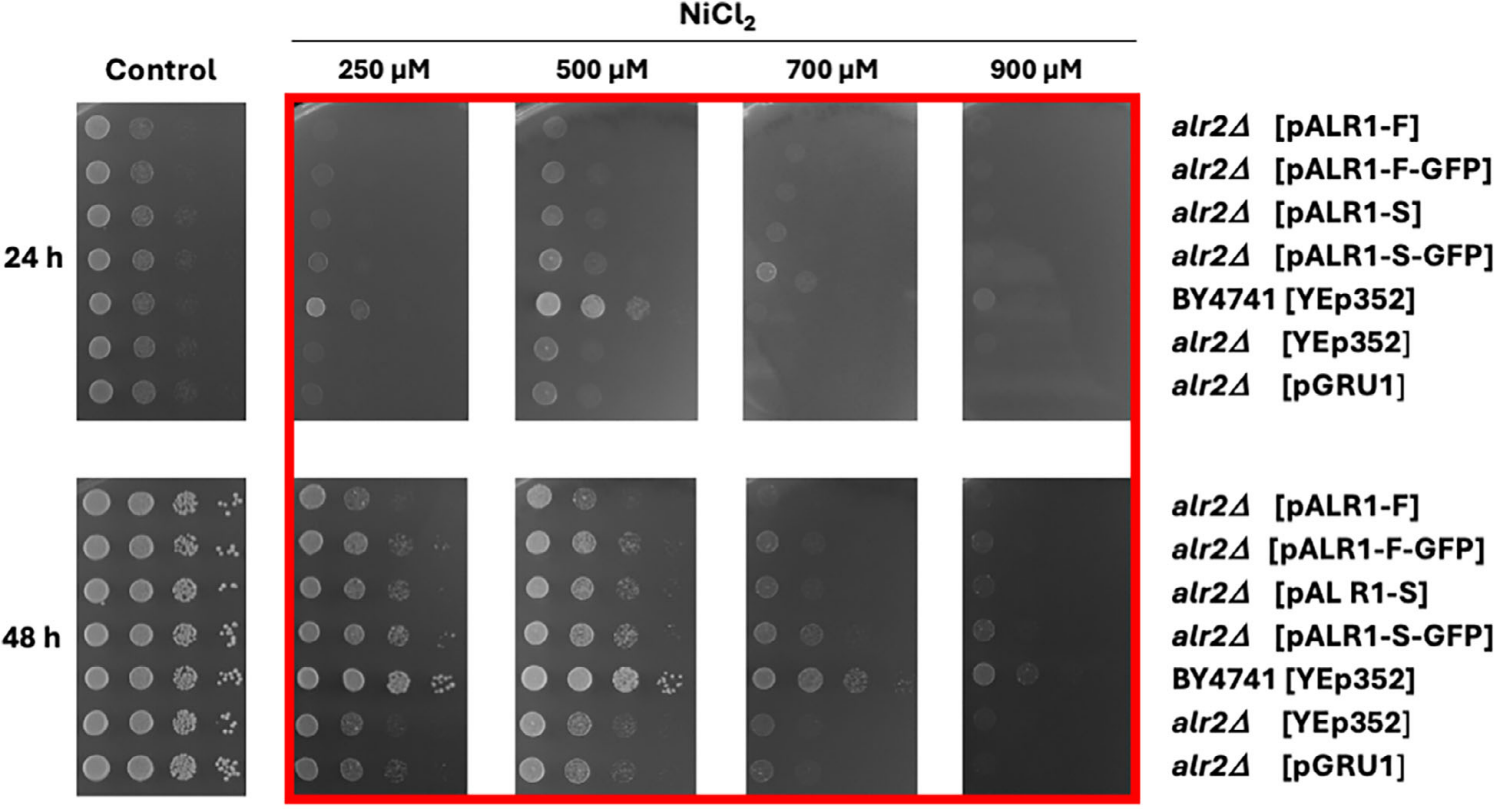
Ni^2+^ tolerance of the wild type or BY4741*alr2Δ* transformants expressing either the empty YEp352 or pGRU1 or plasmids encoding the full or truncated versions of *Pp*Alr1p ± GFP. Cells were grown on YNB media supplemented with Ni^2+^ at indicated concentrations for 24 or 48 h. YNB media without supplies was used as a control.

**FIGURE 6:**
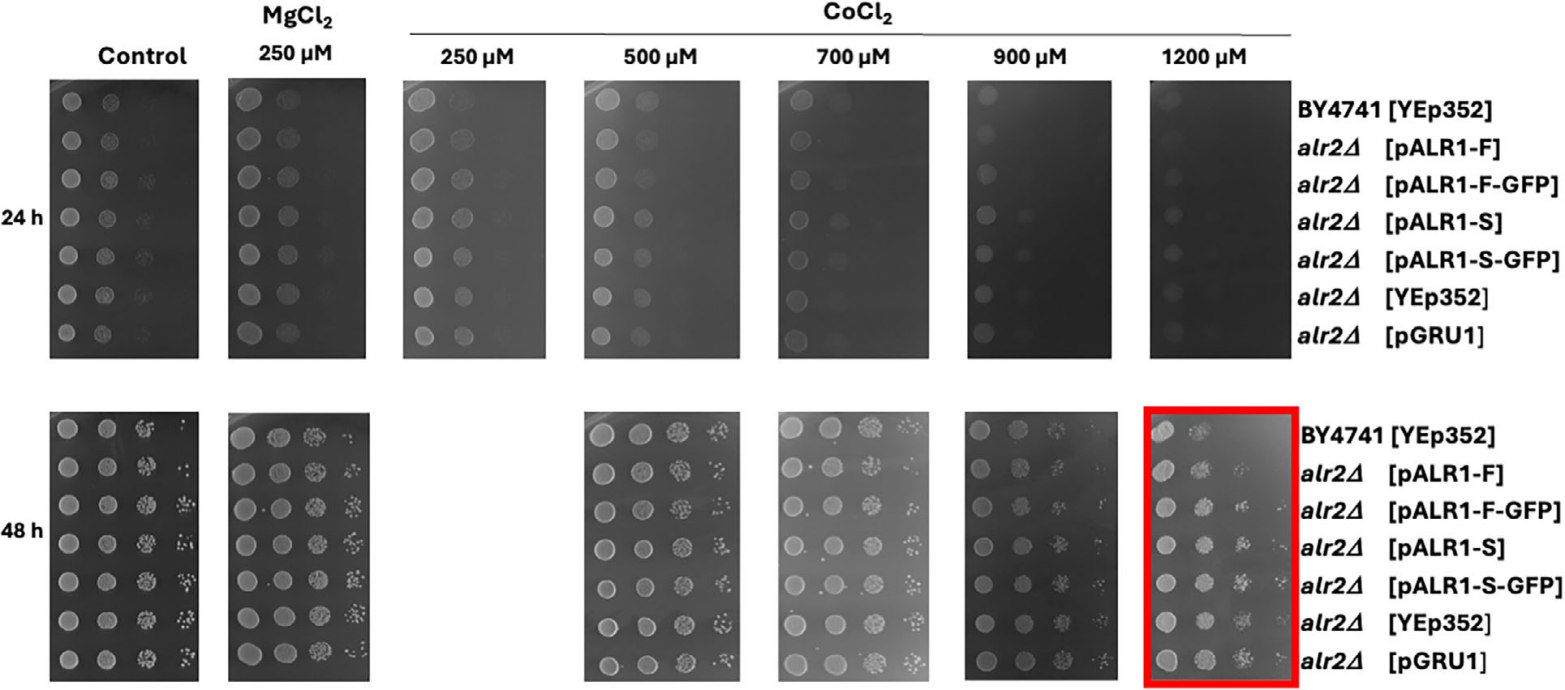
Mg^2+^ and Co^2+^ tolerance of the wild type or BY4741*alr2Δ* transformants expressing either the empty YEp352 or pGRU1 or plasmids encoding the full or truncated versions of *Pp*Alr1p ± GFP. Cells were grown on YNB media supplemented with Mg^2+^ or Co^2+^ at indicated concentrations for 24 or 48 h. YNB media without supplies was used as a control.

**FIGURE 7:**
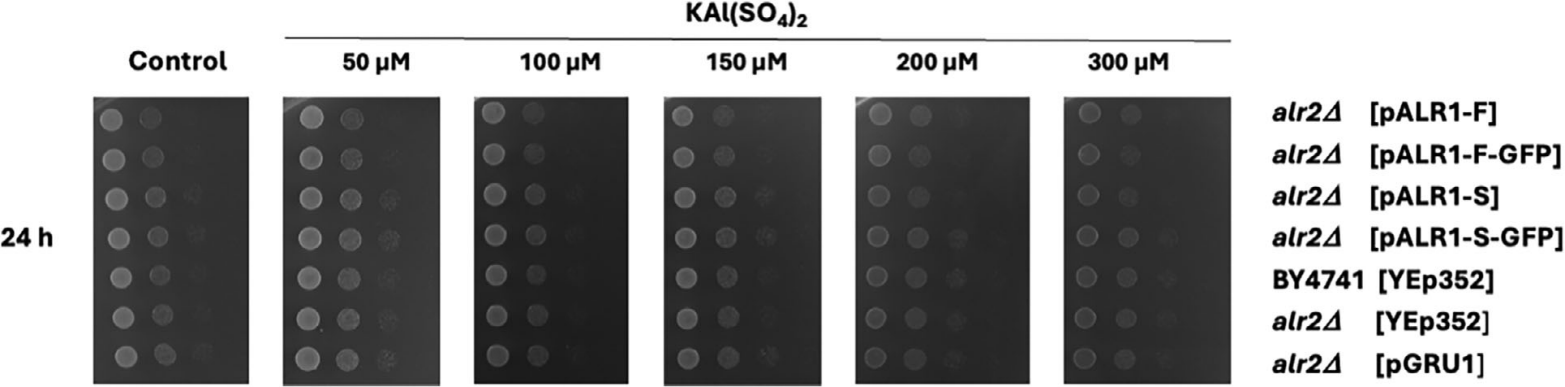
Al^3+^ tolerance of the wild type or BY4741*alr2Δ* transformants expressing either the empty YEp352 or pGRU1 or plasmids encoding the full or truncated versions of *Pp*Alr1 ± GFP. Cells were grown on YNB media supplemented with Al^3+^ at indicated concentrations for 24 h. YNB media without supplies was used as a control.

Per note, *S. cerevisiae* has two *ALR* genes, encoding Alr1 and Alr2 proteins; however, only the *alr2Δ* strain is viable. Therefore, we characterized *S. cerevisiae* strains lacking the *Alr2* gene and expressing the *Pp*Alr1p (full or truncated version) for their tolerance to metal ions. We compared the growth of the wild‐type cells of the *alr2Δ* strain expressing the empty vector (YEp352) or the full or truncated version of *Pp*Alr1p (without or with GFP tagged) on plates supplemented with increasing concentrations of Ni^2+^, Co^2+^, Mg^2+^, and Al^3+^ (Figures 5, 6, 7). Deletion of *ALR2* in *S. cerevisiae* resulted in an increased sensitivity to Ni^2+^, where all *alr2Δ* transformants grew worse than BY4741 without deletion (Figure 5, red square). Possibly, the presence of full‐length *Pp*Alr1p (w/o GFP) even decreases tolerance to Ni^2+^ as the *alr2Δ* cells expressing the full version of *Pp*Alr1p grew slightly worse in the presence of Ni^2+^ than the same cells expressing the truncated version (Figure 5).

Conversely, tolerance has increased for *alr2Δ* strain with or w/o overexpressed *Pp*Alr1p at high Co^2+^ concentrations (Figure 6, red square). The *alr2Δ* transformants grow visibly better than strains expressing both Alr1/2p after 48 h.

The presence of *S. cerevisiae* Alr2 protein (*Sc*Alr2p) (in the wild type cells BY4741) and the full‐length *Pp*Alr1p (both GFP‐tagged and untagged) also had a slightly negative effect on Al^3+^ resistance, but the general effect is barely visible (Figure 7). The cells expressing the short *Pp*Alr1p versions exhibited the same growth in the presence of Al^3+^ as control cells BY4741*alr2Δ* transformed with the empty vectors (Figure 7), but this phenotype is transient and visible only at the early stage of growth.

## 4 DISCUSSION

Alr1/2 proteins are two members of the CorA family responsible for magnesium transport in fungi and are also believed to be involved in aluminum resistance mechanisms (MacDiarmid & Gardner, 1998). Previous reports proposed that *Sc*Alr1/2p participates in the transport of different divalent cations identically to the other CorAs (Kern et al., 2005; Lim et al., 2011; MacDiarmid & Gardner, 1998). However, it remains obscure how *Sc*Alr1/2p can promote Al^3+^‐resistance (MacDiarmid & Gardner, 1998). Whether they merely bind Al^3+^ (and hence inactivate it) or can actively transport Al^3+^ is not known. We and others previously showed that CorAs are in principle semi‐selective with a strong preference for very similar Mg^2+^, Zn^2+^, Co^2+^, and Ni^2+^ (Guskov & Eshaghi, 2012). However, aluminum differs significantly from these divalent ions, with the first hydration shell radius of 1.9 Å versus ~2.1 Å for most of the divalent cations.

Our experiments revealed that the truncated *Pp*Alr1p is fully functional (Figure 3), and most likely represents a functional core unit of the CorA family. However, the extended N‐terminal domain in *Pp*Alr1p is essential for the correct localization of the protein to the plasma membrane (Figure 4). Mechanistically, this domain likely contributes (i) trafficking/ER‐export signals and/or interaction surfaces required for forward transport, and (ii) co‐translational or early post‐translational folding/assembly that passes ER quality control. Consistent with this, the truncated construct accumulates in intracellular puncta, compatible with ER retention, ER exit sites, or aberrant self‐association.

Surprisingly, we were unable to detect any aluminum transport, which can be either a shortcoming of our experimental setup (morin dye) or a result of the truncation. However, in our competitive uptakes, the addition of aluminum stimulates the transport of zinc proportionally to the added aluminum (Figure 3). One of the reasons for such behavior can be the presence of aluminum sensors that stimulate the transport of divalent cations to counteract aluminum toxicity. To verify whether such an aluminum sensing site does exist requires a high‐resolution structure.

Intriguingly, it was shown that the truncation of the N‐terminal domain and the very end of the C‐terminal domain had no effect on the transport of magnesium via *Sc*Alr1p, but further truncation revealed a reduction of growth on low magnesium media (Lee & Gardner, 2006). In addition, the other member of the CorA family which is believed to be affected by Al^3+^ is Mrs2‐10 from *A. thaliana*, which does not possess the large N‐terminal domain. Furthermore, our knockout studies on *Sc*Alr1/2p with the complementation by truncated and full‐length *Pp*Alr1p showed no effect on aluminum tolerance (Figure 7) and increased sensitivity to Ni^2+^ (Figure 5). Although PpAlr1p overexpression might be expected to improve tolerance, our data show decreased tolerance to Ni^2+^ and a modest, early reduction in Al^3+^ resistance. A parsimonious explanation is that PpAlr1p increases cellular entry of divalent cations (notably Ni^2+^), elevating intracellular toxicity. Consistent with this, deletion of *ALR2* unmasks a contribution of PpAlr2p to Ni^2+^ tolerance that PpAlr1p cannot compensate. In addition, Al^3+^ may perturb Mg^2+^ homeostasis under our plate conditions, such that enhanced divalent influx does not translate into improved growth.

Interestingly, despite the previous report showing that the deletion of the flexible end of the C‐terminal domain had no effect on *Sc*Alr1p transport, it affected oligomerization and subcellular allocation of the protein (Lee & Gardner, 2006; Wachek et al., 2006). In stark contrast, during the processing of our EM data we observed pentamers (Figure S1). Whether the exact truncation site is responsible for such differences in the two studies is yet to be investigated.

The obtained structure revealed the pentameric organization of *Pp*Alr1p. A very similar arrangement was observed for other CorAs in a non‐conductive state (Eshaghi et al., 2006; Gati et al., 2017; Guskov & Eshaghi, 2012). Unfortunately, it is impossible to make any meaningful analysis and comparison given the low resolution of the structure we obtained. The presence of magnesium binding sites, as found in prokaryotic CorA (Eshaghi et al., 2006; Guskov & Eshaghi, 2012) in *Pp*Alr1/2p and *Sc*Alr1/2p is yet to be verified. However, an additional supply of magnesium in buffers improved *Pp*Alr1p's stability, indicating that most likely such Mg^2+^‐binding sites are present.

The growth assay of the yeast *alr2Δ* strain with expression of truncated and full versions of *Pp*Alr1p revealed a minor effect on the ability to grow in high concentrations of divalent cations (Figures 5, 6, 7). However, previously, *Sc*Alr2p was assigned a minor role in transport (MacDiarmid & Gardner, 1998), but we observed it has some effect on growth at high concentrations of Ni^2+^ in all the variations of the tested strains (Figure 5, red square). This is in stark contrast to the previously reported negligible effect of *Sc*Alr2p on Ni^2+^ uptake, and all the roles for the transport of Ni^2+^ were assigned to *Sc*Alr1p and *Sc*Mrs2 (Lim et al., 2011). However, in our work, the tolerance to extremely high concentrations of Co^2+^ has increased for all *alr2Δ* transformants (Figure 6, red square), which is in contrast with the previous report, where the *Scalr2Δ* strain was shown to transport Co^2+^ with a higher rate than the wild type (MacDiarmid & Gardner, 1998). Several factors may underlie the observed discrepancy. First, uptake assays and growth tolerance are not directly interchangeable: increased Co^2+^ uptake can sensitize cells, whereas reduced uptake can appear as improved tolerance. Second, strain backgrounds and constructs differ (native *Sc*Alr1/2p versus heterologous *Pp*Alr1p), potentially affecting regulation and stoichiometry. Third, Alr1p and Alr2p are known to hetero‐oligomerize (Wachek et al., 2006); removal of Alr2p may alter selectivity of the pore complex, shifting relative handling of Ni^2+^ and Co^2+^. Finally, tolerance phenotypes integrate multiple processes—transport, buffering, and detoxification—and small differences in these downstream systems may explain divergent outcomes. Together, these considerations suggest that Alr2p plays a modulatory rather than purely redundant role in divalent cation homeostasis.

Interestingly, overexpression of neither truncated nor full‐length *Pp*Alr1p was able to affect the growth in both cases, indicating that *Sc*Alr2p may be more important than previously thought. Further investigations will be necessary to delineate the roles of *Sc*Alr1/2p—preferably with the single deletion of Alr1 (*alr1Δ*) and a double deletion mutant (*alr1Δalr2Δ*).

Furthermore, our functional in vitro characterization of *Pp*Alr1p is incompatible with the previously proposed transport mechanism of cation: proton symport. Our data indicate that the transport is independent from membrane potential and proton gradient (Figure 3). This behavior is more consistent with a passive, facilitated‐diffusion mechanism, in which Alr1p permits divalent cation flux down their concentration gradient without proton coupling. Such a mechanism aligns with the activity of other CorA homologs in reconstituted systems (Stetsenko & Guskov, 2020). An additional possibility is that aluminum acts as an allosteric activator of Alr1p, stimulating divalent cation uptake in a way that bypasses the need for electrochemical driving forces. These alternative mechanisms may explain discrepancies with previous in vivo observations. However, for a more detailed analysis of the transport mechanism and role of *Pp*Alr1p in Al^3+^ tolerance both high‐resolution structural information, electrophysiological characterization and more advanced knock‐out studies are necessary.

## AUTHOR CONTRIBUTIONS

**Pavlo Stehantsev:** Investigation; writing – original draft; visualization; formal analysis. **Artem Stetsenko:** Investigation; writing – original draft; methodology; visualization; formal analysis. **Pavla Herynková:** Investigation; methodology; visualization; formal analysis. **Andrei Zupnik:** Investigation; visualization; writing – review and editing; data curation. **Carsten Takens:** Investigation; formal analysis. **Olga Zimmermannova:** Investigation; writing – review and editing; validation; methodology; formal analysis; supervision. **Hana Sychrova:** Methodology; validation; formal analysis; supervision; resources. **Albert Guskov:** Conceptualization; funding acquisition; writing – original draft; validation; writing – review and editing; formal analysis; project administration; supervision.

## CONFLICT OF INTEREST STATEMENT

The authors declare no conflict of interest.

## Supporting information


**Figure S1.** Data processing of the obtained structure.
**Table S1.** Oligonucleotides used for the *alr2Δ* single deletion in *S. cerevisiae* BY4741.
**Table S2.** Oligonucleotides used for the cloning of long and short versions of *P. pastoris* ALR1 cDNA's into *S. cerevisiae* BY4741 *alr2Δ* construct.

## Data Availability

The data that support the findings of this study are available from the corresponding author upon reasonable request.
