# Supplementary material for: Investigating the mechanism of aluminum resistance conferred by aluminum resistance protein 1
Source: Protein Sci. 2025 Dec 22;35(1):e70405. doi: 10.1002/pro.70405 (PMC12720413; doi:10.1002/pro.70405)
Supplement: Supplementary file 1 — Figure S1. Data processing of the obtained structure. Table S1. Oligonucleotides used for the alr2Δ single deletion in S. cerevisiae BY4741. Table S2. Oligonucleotides used for the cloning of long and short versions of P. pastoris ALR1 cDNA's into S. cerevisiae BY4741 alr2Δ construct. [file PRO-35-e70405-s001.docx]

**Supplementary materials**

**
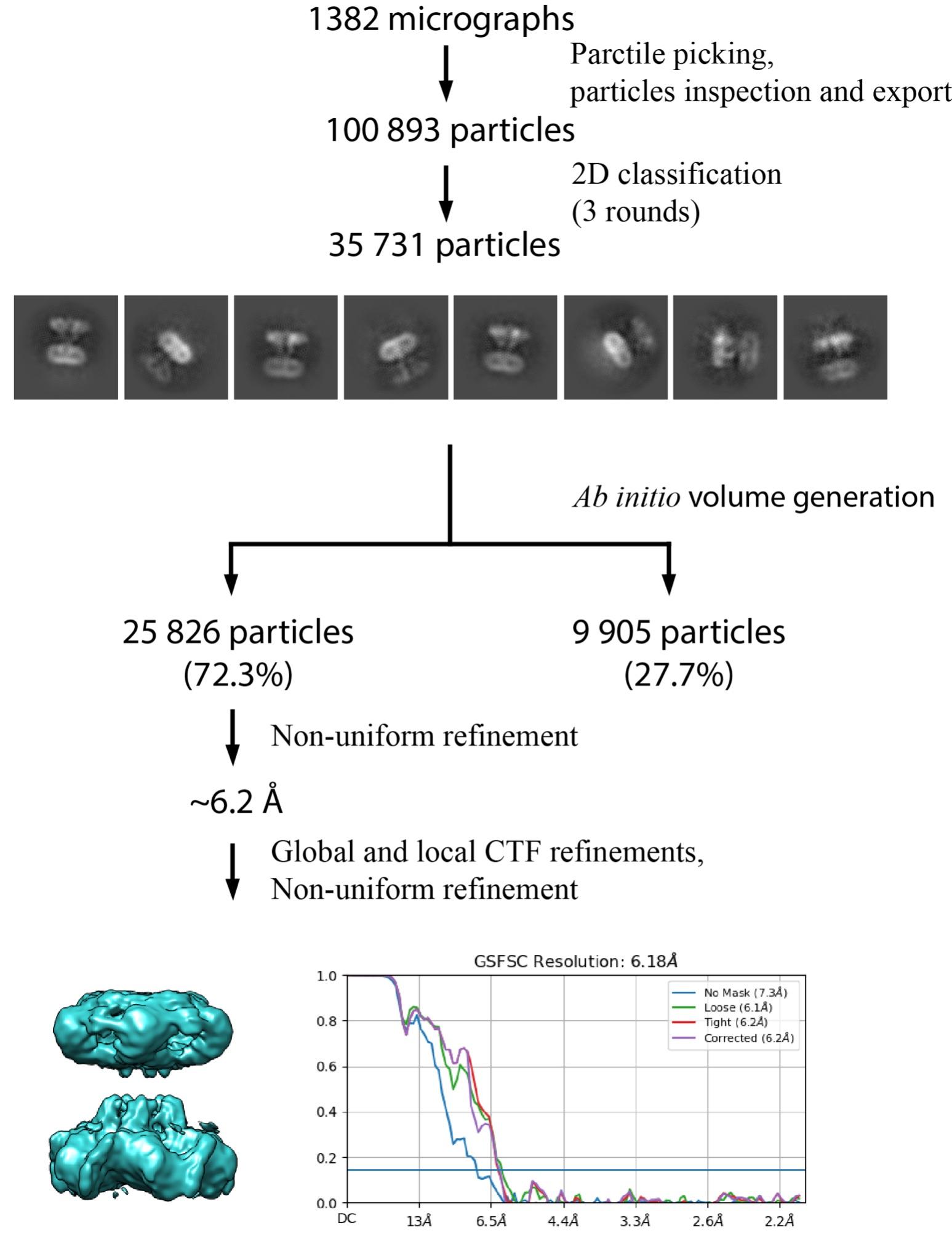
**

Supplementary Figure 1. Data processing of the obtained structure.

Supplementary Table 1. Oligonucleotides used for the *alr2Δ* single deletion in *S. cerevisiae* BY4741

| Oligonucleotide name | Sequence |
| --- | --- |
| ALR2-KAN-F | GAAGTACTGAATTTTTATGAGAAAACGTGAAAAAACTTCGTATTCGTACGCTGCAGGTCGAC |
| ALR2-KAN-R | CTGCCTGCTAAAGATCTGCCGACCTACCATAGCGGTCATGGCATAGGCCACTAGTGGATCTG |
| ALR2-UP-F | CGAAAGTGGCTGCTTCCAG |
| ALR2-R1 | GAGGGAGCGATGCTCTCTAC |
| ALR2-F1 | GGGGGTACTGCTTTTACTAGC |
| ALR2-DOWN-R | GCGCAGTTCCACTGTCAGGC |

Supplementary Table 2. Oligonucleotides used for the cloning of long and short versions of *P. pastoris* ALR1 cDNA’s into *S. cerevisiae* BY4741 *alr2Δ* construct

| Oligonucleotide name | Sequence |
| --- | --- |
| PpALR1-F-F1 | CTCTGAAGATTCTGATAACG |
| PpALR1-R1 | CAACAGCCAACAAACAACCC |
| PpALR1-F2 | CTACTCTTCTGATAACAACG |
| YEpN-PpALR1-F-F | GTACATTATAAAAAAAAATCCTGAACTTAGCTAGATATTATGAGTCCTATCAACGATTCC |
| YEpN-PpALR1-F-R | CACGACGTTGTAAAACGACGGCCAGTGCCAAGCTTGCATGCTAATCGTATTTGGAAAAAGTTC |
| pGRU1N-PpALR1-F-R | TAAAGCTCCGGAGCTTGCATGCCTGCAGGTCGACTCTATCGTATTTGGAAAAAGTTCTTGG |
| YEpN-PpALR1-S-F | GTACATTATAAAAAAAAATCCTGAACTTAGCTAGATATTATGAATGCTGAAGCTGCTTC |
| YEpN-PpALR1-S-R | CACGACGTTGTAAAACGACGGCCAGTGCCAAGCTTGCATGCTAAGTCAAAGTAGTATTAGAAG |
| pGRU1N-PpALR1-S-R | TAAAGCTCCGGAGCTTGCATGCCTGCAGGTCGACTCTAGTCAAAGTAGTATTAGAAGCC |
